# Outcomes of Retesting in Patients with Previously Uninformative Cancer Genetics Evaluations

**DOI:** 10.1007/s10689-021-00276-8

**Published:** 2021-09-21

**Authors:** Shenin A. Sanoba, Erika S. Koeppe, Michelle F. Jacobs, Elena M. Stoffel

**Affiliations:** 1 The Pancreatic Cancer Center at NYU Langone Health, New York, NY; 2 Michigan Medicine Cancer Genetics Clinic, Ann Arbor, MI

**Keywords:** genetic counseling, cancer genetics, hereditary cancer, genetic testing

## Abstract

**Purpose:**

Advances in cancer genetics have increased germline pathogenic/likely pathogenic variant (PV/LPV) detection rates. More data is needed to inform which patients with previously uninformative results could benefit most from retesting, especially beyond breast/ovarian cancer populations. Here, we describe retesting outcomes and predictors of PV/LPVs in a cohort of patients unselected by cancer diagnosis.

**Methods:**

Retrospective chart reviews were conducted for patients at a cancer genetics clinic between 1998-2019 who underwent genetic testing (GT) on ≥2 dates with ≥1 year between tests, with no PV/LPVs on first-line GT. Demographics, retesting indications, and GT details were reviewed to evaluate predictive factors of PV/LPV identification.

**Results:**

139 patients underwent retesting, of whom 24 (17.3%) had a PV/LPV, encompassing 15 genes. 14 PV/LPV carriers (58.3%) only returned for retesting after personal or familial history changes (typically new cancer diagnoses), while 10 (41.7%) retested due to updated GT availability. No specific GT method was most likely to identify PV/LPVs and no specific clinical factors were predictive of a PV/LPV. The identified PV/LPVs were consistent with patients’ personal or family histories, but were discordant with the initial referral indication for GT. For 16 (66.7%) PV/LPV carriers, the genetic diagnosis changed clinical management.

**Conclusion:**

This study adds to the limited body of literature on retesting outcomes beyond first-line *BRCA* analysis alone and confirms the utility of multigene panel testing. Retesting certain affected individuals when updated GT is available could result in earlier PV/LPV identification, significantly impacting screening recommendations and potentially reducing cancer-related morbidity and mortality.

## INTRODUCTION

Germline genetic testing (GT) for hereditary cancer syndromes has evolved rapidly over the past three decades. The genes associated with Hereditary Breast and Ovarian Cancer syndrome (*BRCA1* and *BRCA2*, “*BRCA1/2*”) and Lynch syndrome (*MLH1 MSH2*, *MSH6*, *PMS2*, *EPCAM*) were first identified in the early 1990s.[1, 2] Since tins time, dozens of other genes associated with high- and moderate-penetrance cancer predispositions have also been identified.[2–5]

In addition to new gene discoveries, GT technologies have become more efficient and comprehensive. In the mid-2000s, advances in next generation sequencing (NGS) allowed for the rapid, low-cost assessment of multiple genes simultaneously.[5] The United States Supreme Court’s decision in *Association for Molecular Pathology (AMP) v. Myriad* in June 2013 also impacted GT availability by striking down a patent on the *BRCA1/2* genes. Collectively, advances in NGS and this Supreme Court ruling ultimately resulted in more labs performing GT, a reduction in GT cost, and the expansion of multigene panel testing (MGPT) utilization.[3, 5–7]

There were initial concerns regarding the shift from phenotype-directed testing (i.e., targeted analysis for genes with existing clinical diagnostic or insurance criteria, such as *BRCA1/2*, *TP53*, *APC*, *STK11*, or the Lynch syndrome genes) to MGPT, including higher rates of variants of uncertain significance (VUS), and uncertainty regarding the clinical actionability of findings in less-penetrant genes.[2, 3] However, recent studies have shown that MGPT is time-saving, cost-effective, and improves diagnostic yield.[7–11] Additionally, clinical experience suggests that most reclassified cancer gene VUSs are ultimately benign.[12] Furthermore, cancer risks and clinical guidelines have since been established for many low- to moderate-penetrance genes.[13–17]

Given these GT updates over time, researchers have investigated the outcomes of “retesting,” defined as additional GT after phenotype-directed testing, or reanalysis of the same genes with different technology. Historically, most payers have only covered one round of *BRCA1/2* GT for eligible patients; therefore, most studies focused on retesting after first-line *BRCA1/2* analysis alone (“*BRCA1/2* retesting”).[3, 5, 18–27] The pathogenic/likely pathogenic variant (PV/LPV) detection rate in *BRCA1/2* retesting studies ranged from 3-11% in cohorts unselected for a personal history of cancer, and 5-13% in those with breast or ovarian cancer.[18–27] Few studies so far have examined outcomes of retesting patients whose first-line testing included genes beyond *BRCA1/2.*[28–30]

Existing studies have demonstrated the potential for retesting to diagnose hereditary cancer syndromes , but have also noted disadvantages of retesting with a MGPT. As noted, these include higher VUS rates and uncertainty regarding the clinical actionability of PV/LPVs in some genes, as well as incidental findings unrelated to the observed phenotype, patient out-of-pocket costs, and a prolonged “diagnostic odyssey.”[18–30] As such, clinicians should carefully consider which patients to retest and which genes to include on MGPTs. However, the existing literature does not provide definitive guidance, and retesting protocols have not been clearly defined in the clinical setting.[5, 28, 29, 31, 32] And while recent publications from the American Society of Breast Surgeons and the National Comprehensive Cancer Network briefly comment on the option to retest after limited first-line GT, retesting is not currently mentioned in the genetics- or cancer-related practice guidelines of most major societies and organizations.[4, 13, 31–33]

Further evidence is needed to ascertain the utility of retesting (especially outside of breast and ovarian cancer populations) and to establish which patients to prioritize for retesting. Therefore, we sought to describe the outcomes of retesting in a diverse clinical cohort unselected for particular cancer diagnoses or first-line GT, in order to 1) investigate the outcomes of retesting across the spectrum of hereditary cancer syndromes, and 2) to identity predictors associated with PV/LPV findings upon retesting.

## METHODS

A retrospective database review was conducted for all patients seen in the Michigan Medicine Cancer Genetics Clinic between January 1998 and March 2019 (University of Michigan Cancer Genetics Registry, IRB HUM00043430). This tertiary referral multidisciplinary cancer genetics clinic serves the catchment area of Michigan, northeastern Indiana, and northwestern Ohio. Referrals include a broad range of cancers or non-malignant manifestations, ranging from common diagnoses (e.g., breast and colorectal cancer) to less- common indications (e.g., dermatologic and endocrine cancers).

Patients were eligible for study inclusion if they 1) had germline GT on ≥2 separate dates, with ≥1 calendar year between any 2 rounds of GT, and 2) had no PV/LPVs on first-line GT. “Genetic testing” was defined as germline GT for any gene(s) associated with a hereditary cancer syndrome. Each patient was offered clinical GT based on their personal and/or family history. The genes tested were selected at the clinician’s discretion, with consideration of clinical indication and requirements of the patients’ health insurance; therefore, GT was not standardized across the cohort. Overall, GT typically included sequencing and/or deletion/duplication analysis of 1 - 81 genes performed on a sample of peripheral blood or saliva by a commercial laboratory.

The following data were collected via database and medical record review: sex, race and ethnicity, age at most recent GT, personal history of cancer or non-malignant manifestations warranting a genetics referral (type and age at diagnosis, excluding a history of non-melanoma skin cancers alone), first-line GT indication, reason for retesting (defined in Table 1), genes analyzed, testing method (defined in Table 2), date of GT results, outcome of GT, and impact of GT on clinical care for PV/LPV carriers.

Statistical analysis was performed using the chi-squared test, Fisher’s exact test, or paired t-test (as appropriate) to determine predictors of an informative retest outcome (i.e., PV/LPV). Statistical significance was considered at *p* < 0.05.

## RESULTS

Out of 6556 registry patients seen during the study timeframe, 1880 had no GT; 4537 had one round of GT (PV/LPV identified, *n* = 1347; negative for known familial PV/LPV, *n* = 517; uninformative result, *n* = 2673); and 139 had multiple rounds of GT and met the study inclusion criteria (Online Resource 1). Compared to patients who with uninformative GT who did not retest, this cohort was younger (*p* = <0.001), but no significant differences were observed with respect to sex (*p* = 0.06), personal cancer history (*p* = 0.17), or race and ethnicity (*p* = 0.3).

In the final cohort, 104 (74.8%) patients were affected with a cancer/tumor or other finding warranting GT and 35 (25.2%) were unaffected (Table 3). Both sub-cohorts were predominantly female (*n* = 75, 72.1% affected and *n* = 24, 68.6%, unaffected) and non-Hispanic white (*n* = 98, 94.2% and *n* = 31. 88.6%) . The average age at the most recent GT was 52.6 (range 9-82) for affected patients and 44 (range 5-68) for unaffected patients. Among the affected cohort, the mean age at first diagnosis was 42.5 (range 2-73) and the most common diagnoses were female breast (*n* = 42, 40.4%), cutaneous melanoma (*n* = 27, 26%), and colorectal cancer (*n* = 21, 20.2%) (Table 4). Additionally, 42 (40.4%) had multiple primary diagnoses.

**Figure 1.**
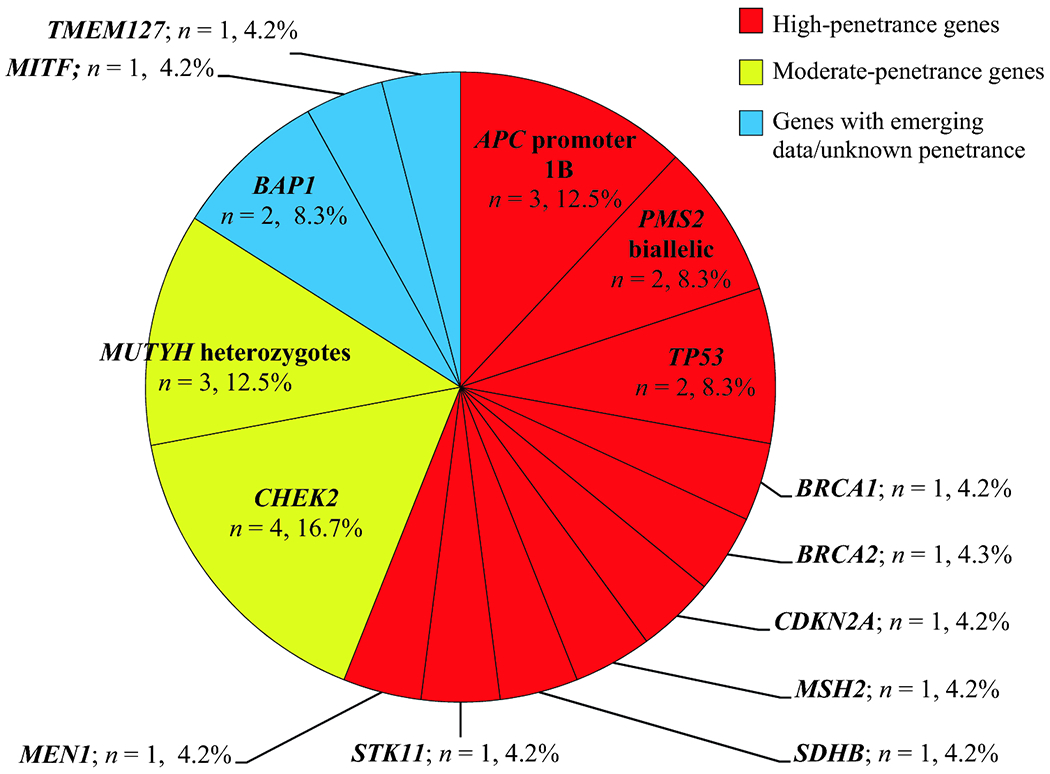
Likely Pathogenic and Pathogenic Variants Identified After Retesting (*n* = 25) Total >100%, as percentages were calculated with regard to *n* = 24 individuals with PVs identified; however, *n* = 25 PVs total, as one patient had 2 PVs (*BRCA1* and *CDKN2A*)

Upon retesting, 24 (17.3%) patients had a PV/LPV, with 21 (87.5%) PV/LPVs identified in affected patients. Overall, 14 (10.1%) patients had a VUS, and 101 (72.6%) had no variants identified (Table 3). A total of 25 PV/LPVs were identified in 15 genes associated with 14 syndromes (Figure 1). 13 PV/LPVs (54.2%) were identified in high-penetrance genes, 7 (29.2%) were found in moderate- or low-penetrance genes, and 4 (16.6%) were in recently-described genes with emerging cancer risk data.

Of 57 patients initially referred for *BRCA1/2* GT, 9 had PV/LPVs identified on retesting, with 7 alterations identified in genes other than *BRCA1/2* (*BAPT CDKN2A*, *CHEK2*, *MSH2*, *MUTYH* heterozygote, *n* = 1 each; *TP53, n* = 2) (Online Resource 2). Of 20 patients initially referred for Lynch syndrome, 3 had PV/LPVs on retesting, and none ultimately had this condition (*CHEK2, MITF, MUTYH* heterozygote, *n* = 1 each). Finally, of 63 patients initially referred for any other syndrome, 13 had PV/LPVs: 2 in breast cancer-related genes (*CHEK2, n* = 2 each), 5 in colon cancer-related genes (*MUTYH* heterozygote, *STK11, n* = 1 each; *APC, n* = 3), and 6 in other cancer genes (*BAP1, MEN1 SDHB*, *TMEM127*, *n* = 1 each; *PMS2* biallelic, *n* = 2). PV/LPVs identified on retesting were largely clinically consistent with the patient’s personal or family history of cancer, but were discordant with the initial, phenotype-directed reason for referral (Online Resource 3).

Among either affected or unaffected patients, no significant difference was observed between PV/LPVs and uninformative results with respect to sex (*p* = 0.46 and 0.54, respectively), age (*p* = 0.37 and 0.43), time between first and most recent GT (*p* = 0.06 and 0.91), or number of rounds of GT (*p* = 0.94 and 0.21) (Table 3). Despite small sample sizes from racial minorities, PV/LPVs were identified in 1 out of 4 (25%) Black/African-American patients, 1 out of 2 (50%) Hispanic patients, 1 out of 3 (33.3%) American Indian/Alaska Native patients, and in the single multiracial, non-Hispanic patient. The likelihood of PV/LPV identification was not statistically significant when affected patients were compared to unaffected patients (*p* = 0.09). The sample sizes for individual cancer types were too small to determine if a particular diagnosis was more likely to result in PV identification (Table 4). However, when considering rare tumor types, PV/LPVs were identified in 3 out of 10 (30%) patients with sarcomas, 2 out of 5 (40%) with ovarian cancer, and the 2 patients with oral squamous cell carcinomas.

Among affected patients, 43 (41.3%) returned for retesting due to a change in their personal and/or family history alone (personal, *n* = 30, 28.8%; familial, *n* = 13, 12.5%), compared to 15 (42.9%) unaffected patients (personal, *n* = 2, 5.7%; familial, *n* = 13, 37.5%), (Table 5). Additionally, 47 (45.2%) affected patients and 20 (57.1%) unaffected patients returned solely due to the availability of updated GT. Some affected patients returned for multiple reasons, including changes to both personal and family history (*n* = 10, 9.6%), or due to updated GT availability and a change in personal (*n* = 1, 1%) or family history (*n* = 3, 2.9%). Of the 40 affected patients who returned due to any personal history change, 24 (60%) returned due to a new diagnosis of cancer or tumor. Among the 24 PV carriers, 14 returned due to a change in their personal history (*n* = 7, 29.2%), family history (*n* = 5, 20.8%), or both (*n* = 2, 8.3%). Of the 7 PV/LPV carriers who sought retesting due to a family history change, 5 had a relative with a PV/LPV identified in the interval since their own first-line, uninformative GT (*BAP1, BRCA2, CHEK2, MITF, MUTYH* heterozygote, *n* = 1 each) (Online Resource 3). Of the 10 (41.7%) PV/LPV carriers who returned due to updated GT, 5 had a prior clinical diagnosis and had variants in concordant genes upon retesting, which were missed on first-line analysis (APC, *n* = 3; *MEN1, STK11, n* = 1 each). In either affected or unaffected patients, identification of PV/LPVs was not significantly correlated with any individual reason for retesting (*p* = 0.6 and 0.62, respectively).

**Figure 2.**
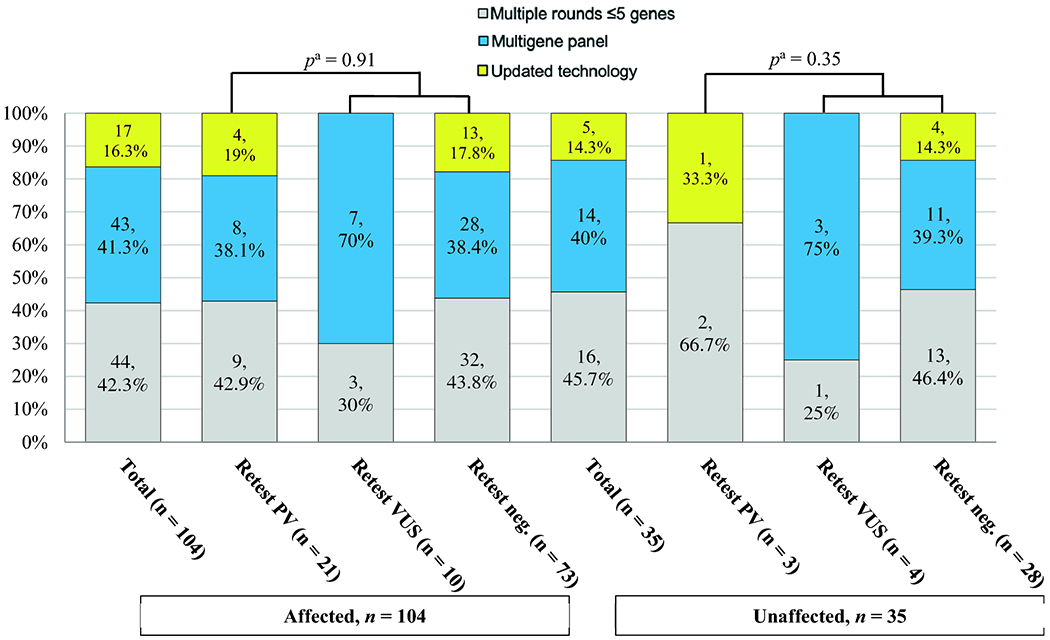
Retesting Methods Utilized *(n* = 139) ^a^Compared informative (positive) results to uninformative results (VUS and negative results collectively).

Overall, the most common retesting method utilized was multiple rounds of syndrome-specific testing (*n* = 44, 42.3% affected patients and *n* = 16, 45.7% unaffected patients, respectively), followed by MGPT (*n* = 43, 41.3% and *n* = 14, 40%) and updated technology (*n* = 17, 16.3% and *n* = 5, 14.3%) (Figure 2). Although no patients were retested with MGPT prior to June 2013 (e.g., time of the *AMP v. Myriad* decision), 57 of the 84 (68%) of patients who presented after this date were retested with MGPT. Of the 24 PV/LPV carriers, 11 (45.8%) were identified via multiple rounds of syndrome-specific testing, 8 (33.3%) with MGPT, and 5 (20.8%) with updated technology (*APC* promoter IB analysis, *n* = 3; *MEN1* deletion/duplication analysis and *STK11* deletion/duplication analysis, *n* = 1 each) (Online Resource 3).

We assessed the clinical impact of retesting on medical care for the 24 PV/LPV carriers and their families through chart reviews (Online Resource 3). Retesting resulted in changes to cancer screening and/or management recommendations for 16 (66.7%) of these patients. Of those with no changes to their medical care, 5 (20.8%) were already followed appropriately due to a clinical diagnosis (*APC, n* = 3; *MEN1, n* = 1; *STK11*, *n* = 1), 2 (8.3%) had metastatic cancer, and 1 (4.2%) already had increased surveillance due to a prior diagnosis (*MUTYH* heterozygote with colon cancer). Additionally, single-site GT was recomnended for all first-degree relatives of PV/LPV carriers identified via retesting.

## DISCUSSION

In this clinical cohort of patients with diverse cancer phenotypes, retesting identified PV/LPVs in approximately 1 out of every 6 individuals with previously uninformative GT, regardless of first-line GT indication. PV/LPVs were identified in 15 genes and 14 syndromes, and were consistent with patients’ personal or family histories (i.e., no secondary or unexpected findings). However, most PV/LPVs were discordant with the initial indication for GT, which was particularly pronounced in patients initially referred for common cancer syndromes. Specifically, the majority of PV/LPV carriers referred for first-line *BRCA1/2* GT ultimately had a variant in a non-*BRCA* gene, and none referred for first-line Lynch syndrome GT ultimately had this condition.

Retesting identified PV/LPVs in 17.3% of our subjects. This exceeds the detection rates of most prior retesting studies, including the 3.3-12.7% PV rate in *BRCA* retesting studies and 5.0-17.6%PV rate in the few existing non-*BRCA* retesting studies.[18–26, 28–30] Relatively few individuals here had a VUS (*n* = 14, 10.0%), compared to the 33-45.5% VUS rates previously reported in non-*BRCA* retesting with MGPT, which may be related to several factors.[28–30] First, prior non-*BRCA* retesting studies included 19-44 genes.[28–30] While the genes retested here ranged from 1-81, most patients were retested for a relatively small number of genes targeted to their personal and family cancer history. Additionally, our study spanned over two decades, and an increased trend of VUS reclassification to benign variant status has been observed with data accumulation over time.[3, 12] Finally, our cohort was comprised of predominantly white patients, while racial and ethnic groups other than non-Hispanic white are more likely to have a VUS—a pervasive and established issue in cancer genetics research.[34]

This data demonstrates that retesting is an effective way to identify PV/LPVs. MGPTs are particularly useful when the differential diagnosis requires consideration of multiple syndromes. MGPTs also increase diagnostic yield for PV/LPVs in syndromes that may be discordant from a phenotype-directed referral, as observed in our cohort.[8, 10, 11] Several PV/LPVs here were found in genes typically only assessed via MGPT, including genes with emerging data regarding cancer risks like *BAP1, TMEM127*, and *MITF*.[14–16]

In the entire cohort, participants were equally likely to return for retesting due to the availability of updated GT, or for a new personal or familial cancer diagnosis. Notably, although some PV/LPV carriers retested due to updated GT availability, the majority were only referred after an interim personal or family history change (23.7% personal; 18.7% familial; 7.2% both)—typically a new cancer diagnosis. Additionally, 5 PV/LPV carriers returned for retesting after a PV/LPV missed in their initial, targeted GT was later identified in a relative, with 3 PV/LPVs found in relatives with interim cancer diagnoses. All PV/LPVs in this study have associated screening or management recommendations; therefore, earlier molecular diagnosis of a hereditary cancer syndrome for the PV/LPV carriers in this study may have provided opportunities for prevention of certain cancers in probands or their relatives.

Collectively, these findings demonstrate that increasing retesting referrals to genetics from oncology professionals based on updated GT availability—rather than on the development of additional cancers—could result in earlier cancer syndrome identification and enable preemptive implementation of screening or risk-reducing measures. Presently, such recommendations have been established or suggested for over 30 genes associated with breast, gynecological, gastrointestinal, endocrine, urological, and dermatological cancer predisposition syndromes.[14–17, 35] Retesting with an up-to-date, comprehensive MGPT could enhance PV/LPV detection across the spectrum of hereditary cancer syndromes.

Despite the potential for retesting to reduce cancer-related morbidities and mortalities, several barriers exist to this process. First, in subjects with uninformative *BRCA1/2* GT, concerns regarding out-of-pocket cost deterred patients from retesting, despite considerable interest.[7, 9, 32, 36] Historically, most payers only covered one round of GT for specific highly-penetrant genes and denied MGPT coverage due to uncertainty regarding the clinical validity and utility of findings in other genes.[3, 5, 9] While such concerns may have been valid immediately post-*AMP v. Myriad*, data regarding cancer risks for dozens of other genes has since emerged and clinical screening guidelines have been established.[3, 7, 9, 12, 17, 35] Additionally, it is now clear that MGPT conserves temporal, financial, and clinical resources while improving diagnostic yield.[3, 8, 9, 17, 35, 37] Establishing MGPT coverage—especially after limited first-line GT—would eliminate a substantial retesting issue. However, further studies are needed to determine which genes to include on MGPTs in various retesting scenarios in order to maximize clinical utility.

Another barrier involves retesting logistics. In busy clinics with limited resources, it is not feasible to retest all patients with uninformative first-line GT, or to continually recontact patients regarding updated GT availability.[38] As such, one goal of this study was to identify whether any specific factors were predictive of PV identification. Although no individual variables were definitively predictive, affected individuals are generally more informative GT candidates than unaffected subjects; indeed, 87.5% of the PV/LPVs in this study were found in affected patients.[35] Prior studies have also straggled to define whom to retest, but suggested prioritizing affected patients; yet these focused almost exclusively on *BRCA1/2* retesting in breast and ovarian cancer populations, while our study adds to the limited body of literature on retesting outcomes in individuals with other cancer types.[11, 18–30] While the sample sizes here were not large enough to define specific cancers or non-malignant manifestations that warrant retesting, further research will be critical to delineate which subsets of affected patients derive the most benefit from retesting, especially outside of *BRCA1/2*-associated cancers.

Several actions could increase awareness of retesting availability. When disclosing first-line GT results, genetic counselors could encourage patients to periodically recontact the clinic regarding updated GT.[38, 39] Prior studies on patients with uninformative *BRCA1/2* GT found that they were more likely to return to clinic if informed that retesting could help their relatives; therefore, emphasizing potential benefits to family could increase retesting uptake.[36, 40] Additionally, communicating that clinical GT is now more affordable (e.g., minimum $250 out-of-pocket cost in 2021) may be helpful.[36, 37, 40] Genetic counselors can also mention retesting availability in outreach efforts to physicians, and information on retesting should be included in medical school courses or continuing education units on cancer genetics. Finally, although retesting is not currently mentioned in the practice guidelines of most genetics- or oncology-focused professional organizations, further studies to assess the utility of retesting across hereditary cancer syndromes may eventually produce sufficient data to update these guidelines, ultimately increasing physician awareness and retesting referrals.[4, 13, 31–33]

We acknowledge this study had certain limitations. First, the cohort was derived from a tertiary referral specialty genetics clinic comprised of patients with a personal and/or family histories of cancer which warranted multiple genetics referrals, which may not be representative. Second, our sample size was likely too small to identify predictors of which patients were most likely to ultimately have informative retesting results. Finally, the types of GT performed were not standardized across the cohort, and some PVs may have been missed. In a cohort unselected for personal history of cancer, tumor type, or specific gene analyzed on first line GT, PV/LPVs were identified in 17.3% of individuals after additional GT. This work demonstrates the utility of retesting and MGPT across a broad range of hereditary cancer syndromes.

Overall, in a cohort unselected with regard to personal cancer history or type of first-line GT, 17/3% had PV/LPVs identified after additional GT, demonstrating the utility of retesting across a range of hereditary cancer syndromes. Most PV/LPV carriers were only retested after an interim change to their personal or family history (typically a new cancer diagnosis). All identified PV/LPVs were in genes with available screening or management guidelines. The molecular diagnosis changed clinical care for 66.7% of PV/LPV carriers and enabled cascade testing for relatives. Increasing retesting referrals simply due to updated GT availability (rather than new cancer diagnoses) has the potential to result in earlier PV/LPV identification, impacting clinical care and potentially reducing cancer-related morbidities and mortalities for probands and their families. Further research is necessary to determine which clinical factors warrant stronger consideration for retesting.

## Supplementary Material

1750435_OL_1

1750435_OL_2

1750435_OL_3

## Figures and Tables

**Table 1.** Reasons for Retesting

Category	Definition
**Changes to personal history**	• Diagnosis of cancer or other non-malignant manifestations (e.g., polyposis, primary hyperparathyroidism) associated with hereditary cancer syndromes in the proband after first GT
**Changes to family history**	• Diagnosis of cancer or identification of a PV/LPV in a relative after proband’s first-line GT
**Availability of updated GT**	• Availability of updated technology to re-analyze previously tested genes (e.g., deletion/duplication analysis after prior sequencing and 5-site rearrangement analysis of *BRCA1/2*) OR • Availability of MGPT after previous syndrome-specific GT (e.g., first-line testing for only *BRCA1/2* or Lynch syndrome)

**Table 2.** Retesting Methods

Category	Definition
**Multiple rounds of syndrome-specific testing**	• Multiple rounds of GT, with ≤5 syndrome-specific gene(s) tested each time^a,b^
**Multigene panel**	• Analysis of ≤5 syndrome-specific gene(s)^b^, followed by subsequent analysis of any >5 genes
**Updated technology**	• Multiple rounds of GT for the same gene, with different technology utilized upon subsequent analysis (e.g., sequencing followed by deletion/duplication analysis, analysis for founder variants followed by comprehensive gene analysis, *APC* promoter 1B deletion analysis)

a Threshold of ≤5 genes selected to account for analysis of Lynch syndrome genes alone.

b “Syndrome-specific testing” excludes MGPT.

**Table 3.** Cohort Demographics, Personal History, and Retesting Information (*n* = 139)

	Affected Subset^a^					Unaffected Subset				

	Total (*n* = 104)	Retest PV (*n* = 21, 20.2%)	Retest VUS (*n* = 10, 9.6%)	Retest Negative (*n* = 73, 70.2%)	*p* ^b^	Total (*n* = 35)	Retest PV (*n* = 3, 8.6%)	Retest VUS (*n* = 4, 11.4%)	Retest Negative (*n* = 28, 80%)	*p* ^b^

**Female**	75 (72.1%)	17 (81%)	8 (80%)	50 (68.5%)	0.46	24 (68.6%)	3 (100%)	2 (50%)	19 (67.9%)	0.54

**Race, Ethnicity** ^c^					0.19					0.73
White, NH	98 (94.2%)	18 (85.7%)	8 (80%)	72 (98.6%)		31 (88.6%)	2 (66.7%)	3 (75%)	26 (92.9%)	
White, Hispanic	0	0	0	0		2 (5.7%)	1 (33.3%)	0	1 (3.6%)	
Black/African-American, NH	3 (2.9%)	1 (4.8%)	2 (20%)	0		1 (2.9%)	0	1 (25%)	0	
American Indian/Alaska Native, NH	2 (1.9%)	1 (4.8%)	0	1 (1.4%)		1 (2.9%)	0	0	1 (3.6%)	
Multiracial, NH	1 (1%)	1 (4.8%)	0	0		0	0	0	0	

**Mean age at last round of testing, years (range)**	52.6 (9 – 82)	49.9 (19 – 73)	49.2 (9 – 80)	53.8 (12 – 82)	0.37	44 (5 – 68)	52.7 (46 – 59)	40.5 (34 – 52)	43.5 (5 – 68)	0.43

**Mean time, initial test to retest, years (range)**	5.2 (1.1-13.3)	6.4 (1.1-13.3)	5.6 (2.8-7.7)	5.2 (1.1-13.1)	0.06	5.6 (1.2-11.9)	5.4 (2.3-8.6)	6.9 (3-11.9)	5.5 (1.2-10.6)	0.91

**Mean number of rounds of GT ordered (range)**	3 (2-6)	3 (2-5)	2 (2-3)	3 (2-6)	0.94	2 (2 – 4)	2	3 (2 – 4)	3 (2 – 4)	0.21

*NH*, non-Hispanic.

a Includes non-malignant tumors and individuals with a clinical diagnosis of a hereditary cancer predisposition syndrome.

b Compared informative (positive) results to uninformative results (VUS and negative results collectively).

c In calculating *p*-value, compared non-Hispanic white individuals to all other ethnic/racial groups.

**Table 4.** Cancer Diagnoses and Non-Malignant Manifestations in Affected Patients (*n* = 104)

	Retest PV, *n* = 21 (20.2%)	Retest VUS, *n* = 10 (9.6%)	Retest Negative, *n* = 73 (70.2%)	*p* ^a^

**Mean age at first diagnosis; years, (range)** Total: 42.5 (2-73)	38.9 (11-73)	35.8 (5-53)	44.5 (2-69)	0.26

**≥2 primary diagnoses** Total: *n* = 42 (40.4%)	8 (19%)	6 (14.3%)	28 (66.7%)	0.10

**Cancer type/non-malignant manifestation** ^b,c^				
Female breast (*n* = 42, 40.4%)	8 (19%)	3 (7.1%)	31 (73.8%)	>0.05
Melanoma (*n* = 27, 26%)	3 (11.1%)	2 (7.4%)	22 (81.5%)	
Colorectal (*n* = 21, 20.2%)	2 (9.5%)	2 (7.4%)	22 (81.5%)	
Other^d^ (*n* = 12, 11.5%)	4 (33.3%)	2 (16.7%)	16 (76.2%)	
Sarcoma (*n* = 10, 9.6%)	3 (30%)	0	7 (70%)	
PGL/PCC (*n* = 9, 8.7%)	2 (22.2%)	1 (11.1%)	6 (66.7%)	
Thyroid (*n* = 8, 7.7%)	1 (12.5%)	1 (12.5%)	6 (75%)	
Endometrial (*n* = 6, 5.8%)	2 (33.3%)	0	4 (66.7%)	
Ovarian (*n* = 5, 4.8%)	2 (40%)	2 (40%)	1 (20%)	
Polyposis^e^ (*n* = 5, 4.8%)	3 (60%)	0	2 (40%)	
Genitourinary (*n* = 3, 2.9%)	0	0	3 (100%)	
Leukemia (*n* = 3, 2.9%)	0	0	3 (100%)	
ACC (*n* = 3, 2.9%)	0	0	3 (100%)	
Oral SCC (*n* = 2, 1.9%)	2 (100%)	0	0	
Brain (*n* = 2, 1.9%)	1 (50%)	1 (50%)	0	
HL (*n* = 2, 1.9%)	1 (50%)	1 (50%)	0	
Hypercalcemia (*n* = 2, 1.9%)	1 (50%)	1 (50%)	0	
Male breast (*n* = 2, 1.9%)	0	0	2 (100%)	
Prostate (*n* = 2, 1.9%)	0	0	2 (100%)	
Lung (*n* = 2, 1.9%)	0	0	2 (100%)	

*ACC*, adrenocortical carcinoma; *HL*, Hodgkin’s lymphoma; *PGL/PCC*, paraganglioma or pheochromocytoma; *SCC*, squamous cell carcinoma.

a Compared informative (positive) results to uninformative results (VUS and negative results collectively).

b Includes non-malignant tumors associated with hereditary cancer predisposition syndromes and individuals with a clinical diagnosis of a hereditary cancer predisposition syndrome.

c Total *n* >104 and >100%, as *n* = 42 had multiple primary cancers/non-malignant manifestations. Additional diagnoses of non-melanoma skin cancers alone were excluded.

d “Other” encompasses any cancer/non-malignant manifestation only observed once. Retest PV: gastrinoma, pancreatic neuroendocrine tumor, and primary hyperparathyroidism (*n* = 1 each, all in same patient); peritoneal mesothelioma (*n* = 1). Retest VUS: ovarian granulosa cell tumor, retinal hemangioblastoma (*n* = 1 each). Retest negative: bilateral acoustic neuromas, small bowel carcinoid, parotid gland tumor, sebaceous neoplasm; pancreatic (*n* = 1 each).

e Clinical diagnosis of a hereditary polyposis syndrome. Retest PV: Familial Adenomatous Polyposis (*n* = 2) and Peutz-Jeghers syndrome (*n* = 1). Retest negative: Familial Adenomatous Polyposis and Juvenile Polyposis syndrome (*n* = 1 each).

**Table 5.** Reasons for Retesting (*n* = 139)

	Affected Subset^a^					Unaffected Subset				

	Total (*n* = 104)	Retest PV (*n* = 21)	Retest VUS (*n* = 10)	Retest Negative (*n* = 71)	*p* ^b^	Total (*n* = 35)	Retest PV (*n* = 3)	Retest VUS (*n* = 4)	Retest Negative (*n* = 30)	*p* ^b^

**Changes to personal history** ^c,d^	30 (28.8%)	7 (33.3%)	4 (40%)	19 (26%)	0.6	2 (5.7%)	0	1 (25%)	1 (3.6%)	0.62
	
**Changes to family history**	13 (12.5%)	3 (14.3%)	1 (10%)	9 (12.3%)		13 (37.1%)	2 (66.7%)	0	11 (39.3%)	
	
**Availability of updated GT** ^e^	47 (45.2%)	9 (42.9%)	4 (40%)	34 (46.6%)		20 (57.1%)	1 (33.3%)	3 (75%)	16 (57.1%)	
	
**Multiple reasons**										
Changes to personal and family history^c^	10 (9.6%)	2 (9.5%)	1 (10%)	7 (9.6%)		0	0	0	0	
Changes to personal history and updated GT^c,e^	1 (1%)	0	0	1 (1.4%)		0	0	0	0	
Changes to family history and updated GT^e^	3 (2.9%)	0	0	3 (4.1%)		0	0	0	0	

a Includes non-malignant tumors and individuals with a clinical diagnosis of a hereditary cancer predisposition syndrome.

b Compared informative (positive) results to uninformative results (VUS and negative results collectively).

c Among affected patients, changes to personal history included: new cancer or non-malignant tumor/manifestation, *n* = 24; development of additional colon polyps, *n* = 8; other change, *n* = 6; recurrence or metastatic disease, *n* = 2.

d Two patients with no personal cancer history and no clinical diagnosis of a known cancer predisposition syndrome; referred back due to the development of numerous additional colon polyps (*n* = 1) and the development of primary hyperparathyroidism (*n* = 1), respectively.

e Availability of updated GT further defined as: referred back by non-genetics provider, *n* = 11 affected and *n* = 18 unaffected, respectively; patient-initiated return to clinic, *n* = 4 and *n* = 3; unknown, *n* = 5 affected; clinical confirmation of a PV identified via research, *n* = 2 affected; retested during routine follow-up with genetics clinic, *n* = 1 affected; and recontacted by genetics clinic, *n* = 1 affected.

## Data Availability

To protect the privacy of the participants, data regarding this clinical cohort is not publicly available. A de-identified version of the dataset used in this study is available upon request from the corresponding author, S.A.S.
