# Supplementary material for: Outcomes of Retesting in Patients with Previously Uninformative Cancer Genetics Evaluations
Source: Fam Cancer. Author manuscript; Available in PMC 2023 Jul 1. (PMC8934750; doi:10.1007/s10689-021-00276-8)
Supplement: 1750435_OL_1 [file NIHMS1750435-supplement-1750435_OL_1.pdf]

Online Resource 1. Comparison of Patients with Previously Uninformative Results Who Returned for Retesting vs. Patients Who Did Not Return

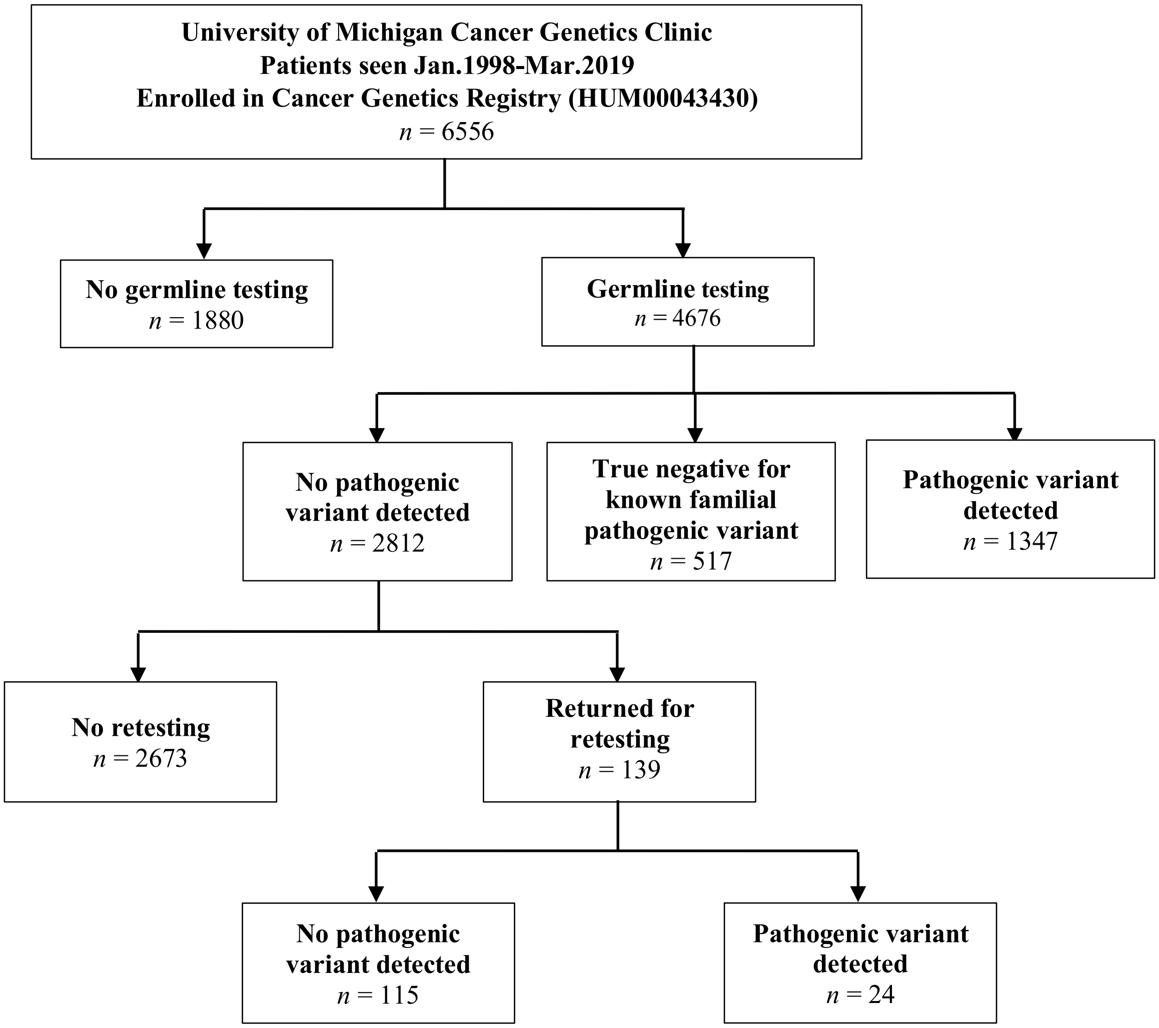

	No retesting<br>*n* = 2673	Returned for retesting<br>*n* = 139	*p*[a]
**Mean age at initial testing, years (range)**	51.1 (0-89)	45.1 (range 1-74)	<0.001
Female	1693 (63.3%)	99 (71.2%)	0.06
**Personal history of cancer**	1208 (54.2%)	71 (51.1%)	0.17
**Race, ethnicity**			
White, NH	2410 (90.2%)	129 (92.8%)	
White, Hispanic	21 (0.79%)	2 (1.4%)	
Black/AA, NH	99 (3.7%)	4 (2.9%)	
Asian	40 (1.5%)	0 (0.0%)	0.3
American Indian/Alaska Native, NH	9 (0.34%)	3 (2.2%)	
Multiracial, NH	14 (0.52%)	1 (0.7%)	
Other/unknown	80 (3.0%)	0 (0.0%)	

AA, African-American; *NH*, non-Hispanic.
[a]In calculating *p*-value, compared non-Hispanic white individuals to all other ethnic/racial groups.

Outcomes of Retesting in Patients with Previously Uninformative Cancer Genetics Evaluations, *Familial Cancer.*
Shenin A. Dettwyler, Erika S. Koeppe, Michelle F. Jacobs, and Elena M. Stoffel
