# Supplementary material for: Outcomes of Retesting in Patients with Previously Uninformative Cancer Genetics Evaluations
Source: Fam Cancer. Author manuscript; Available in PMC 2023 Jul 1. (PMC8934750; doi:10.1007/s10689-021-00276-8)
Supplement: 1750435_OL_2 [file NIHMS1750435-supplement-1750435_OL_2.pdf]

Online Resource 2. Discordance Between Initial Referral Indication and Final Genetic Testing Outcomes (*n* = 139)

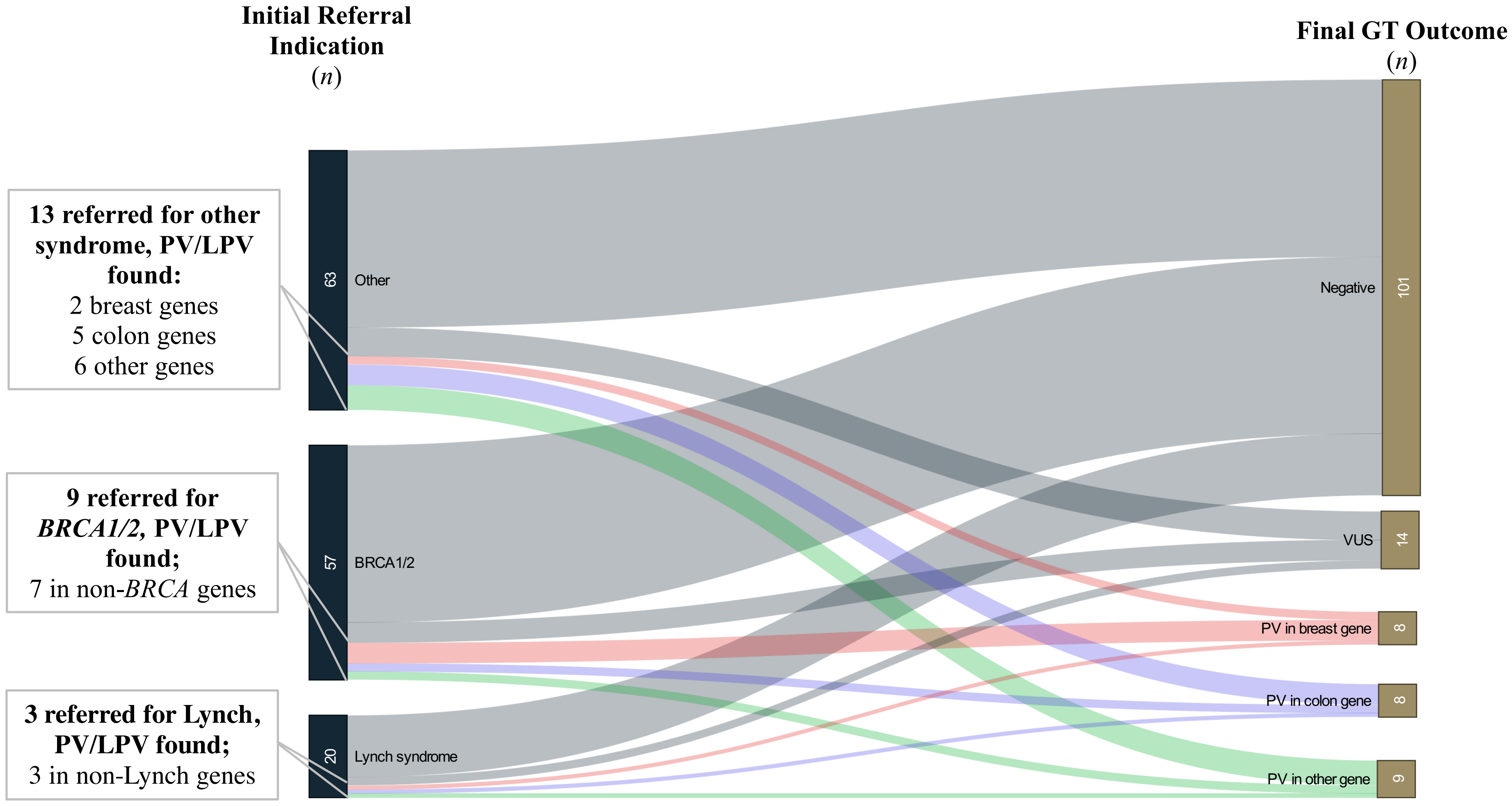

Source: Sankey Diagram Generator V1.2 – Acquire Procure.
Outcomes of Retesting in Patients with Previously Uninformative Cancer Genetics Evaluations, *Familial Cancer.*
Shenin A. Dettwyler, Erika S. Koeppe, Michelle F. Jacobs, and Elena M. Stoffel
